# Application of combinatorial optimization strategies in synthetic biology

**DOI:** 10.1038/s41467-020-16175-y

**Published:** 2020-05-15

**Authors:** Gita Naseri, Mattheos A. G. Koffas

**Affiliations:** 1 Institut für Chemie, Humboldt Universität zu Berlin, 12489 Berlin, Germany; 2 Center for Biotechnology, Rensselaer Polytechnic Institute, Troy, NY USA; 3 Department of Chemical and Biological Engineering, Rensselaer Polytechnic Institute, Troy, NY USA; 4 Department of Biological Sciences, Rensselaer Polytechnic Institute, Troy, NY USA

**Keywords:** Metabolic engineering, Synthetic biology, Machine learning, CRISPR-Cas9 genome editing

## Abstract

In the first wave of synthetic biology, genetic elements, combined into simple circuits, are used to control individual cellular functions. In the second wave of synthetic biology, the simple circuits, combined into complex circuits, form systems-level functions. However, efforts to construct complex circuits are often impeded by our limited knowledge of the optimal combination of individual circuits. For example, a fundamental question in most metabolic engineering projects is the optimal level of enzymes for maximizing the output. To address this point, combinatorial optimization approaches have been established, allowing automatic optimization without prior knowledge of the best combination of expression levels of individual genes. This review focuses on current combinatorial optimization methods and emerging technologies facilitating their applications.

## Abstract (web summary)

Our efforts to build complex synthetic biology circuits are impeded by limited knowledge of optimal combinations. In this review, the authors consider current combinatorial methods and look to emerging technologies.

## Introduction

In the past decade, synthetic biologists have built an impressive collection of elements and tools (genetic sequences performing defined functions such as promoters) and combined them to achieve circuits with more advanced functions e.g. transcriptional regulation. These circuits are now being combined to design regulatory circuits with complex performance, such as logical gates^1^, RNA riboswitches^2,3^, oscillators^4^, and recorders^5^.

However, despite great developments of cutting-edge synthetic biology tools, engineering microorganisms for industrial scale production is still a challenging effort, even for well-known metabolic pathways^6^. Often, multiple genes must be introduced into the host and expressed at appropriate levels to achieve the best possible output. Due to the enormous complexity of living cells, it is typically unknown at which level heterologous genes must be expressed, and to which level the expression of host-endogenous genes must be altered (if not deleted), to accomplish the goal^7^. Therefore, synthetic biologists aim to develop computational tools that can allow prediction of the performance of an assembly or an entire recombinant microorganism^8,9^. However, results from the computational analysis typically require validation through further wet-lab testing. This difficulty principally stems from the nonlinearity of biological systems^10^ and low-throughput characterization methods^11^. Furthermore, it is not always clear how to control noise^12^ and how to transfer the functionality of engineered elements between organisms^7^. Moreover, tweaking multiple factors can typically be critical to obtain an optimal output in a biological system^13^. Those may include overall structural state of chromatin and its domains^14^, the strength of transcriptional regulators controlling gene expression^8,15^, transcriptional terminators^16–18^, ribosome binding sites (RBS), biochemical properties of the protein(s) encoded by the recombinant genes^19–21^, the availability of cofactors for the correct functionality of enyzmes^22,23^, the genetic background of the host^24–26^, and the expression system itself (plasmid-based vs. chromosomal integration)^27^.

To overcome these issues, two types of optimization strategies are available. The first one is “sequential optimization”, a classic method to optimize pathway performance^28^. The sequential flux maximization methodologies frequently utilize deletion of genes encoding competing pathways^29^. However, deletion of a gene can have broad physiological consequences that decrease cellular growth and productivity. For example, different levels of ArgR downregulation, achieved by CRISPR interference (CRISPRi), resulted in two times higher growth rates of *Escherichia coli* compared to deletion of ArgR^30^. However, increasing the production rate of a heterologous product in a recombinant microorganism is complicated and narrowing the research to debottlenecking strategies is too much of a simplification. For example, extensive work has been conducted to investigate the metabolism of the budding yeast *Saccharomyces cerevisiae*; nonetheless, there is still little progress in industrial scale production of high-value chemicals in this organism^31^. In one example, 244,000 synthetic DNA sequences were recently designed to uncover design principles of optimized translation in the well-known prokaryotic host *E. coli*^32^. Although impressive, this work provided little information about possible mechanisms underlying the improved translation capacity.

**Fig. 1:**
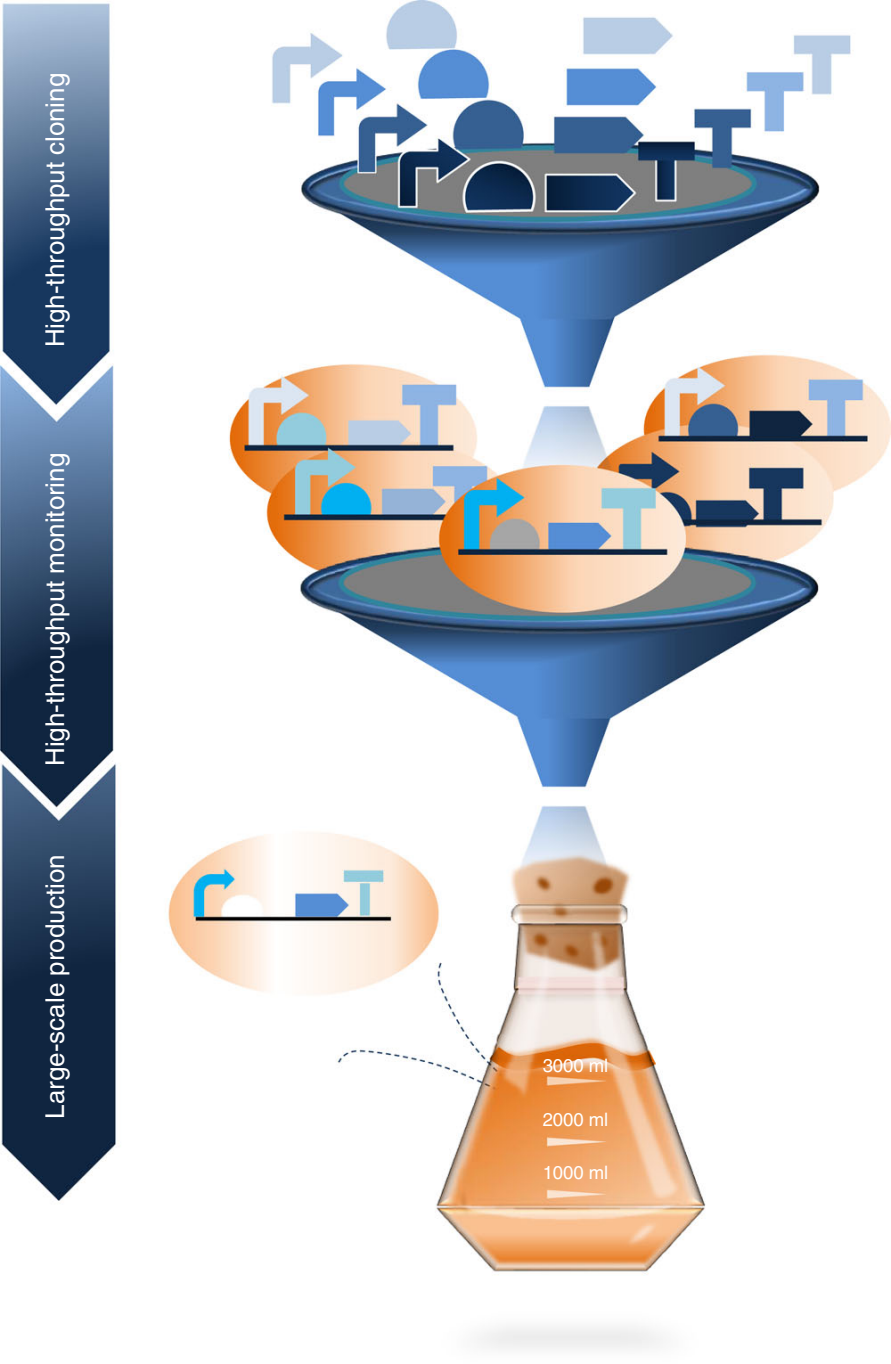
Schematic workflow for microbial factory optimization. Libraries of pathway elements such as promoters (bent arrow), RBSs (chord), coding sequences (arrow), terminators (“T”) are assembled to generate a combinatorial library, in which the microbial members produce different levels of the target metabolite. High-throughput techniques screen the library for the optimized pathway variant. Consequently, the best producer is used for large-scale production.

Using sequential optimization, only one part, or a small number of parts, is tested at a time, making the approach time-consuming and expensive^12,33^ and successful engineering of pathways is usually achieved only by trial-and-error^7^. Another approach to circumvent these barriers is to establish pathway “optimization” methods that do not require prior knowledge of the optimal expression levels of each individual gene involved in a multi-enzyme pathway. Several such methods have recently been developed, such as the functional optimization of gene clusters^34^, perturbation of the global transcription machinery^35^, genomic-scale mapping of fitness modifying genes^36^, multiplex automated genome engineering^37,38^, and “combinatorial optimization”. Jeschek et al. defined combinatorial optimization as “multivariate optimization” (in the context of metabolic engineering)^7^. The combinatorial optimization allows the rapid generation of a large number of diverse genetic constructs in short time^7^. Later on, to achieve high-level production of metabolites, microbial strains in a library that produce the highest level of a metabolite of interest need to be identified (Fig. 1)^39,40^.

Here, we present recent advances in synthetic biology tools that enable the development of complex libraries, and we summarize combinatorial optimization strategies that have been developed recently. Next, we discuss the application of barcoding tools to facilitate tracking diversity to streamline combinatorial optimization techniques. Furthermore, we discuss the application of “biosensors” for high-throughput screening used within the frame of combinatorial optimization. We highlight development in computational and machine learning methods to help generate optimal constructs through minimizing or maximizing target functions out of a defined subject. Finally, we outline applications of combinatorial pathway optimization methods beyond metabolic engineering.

## From combinatorial optimization to efficient production

**Fig. 2:**
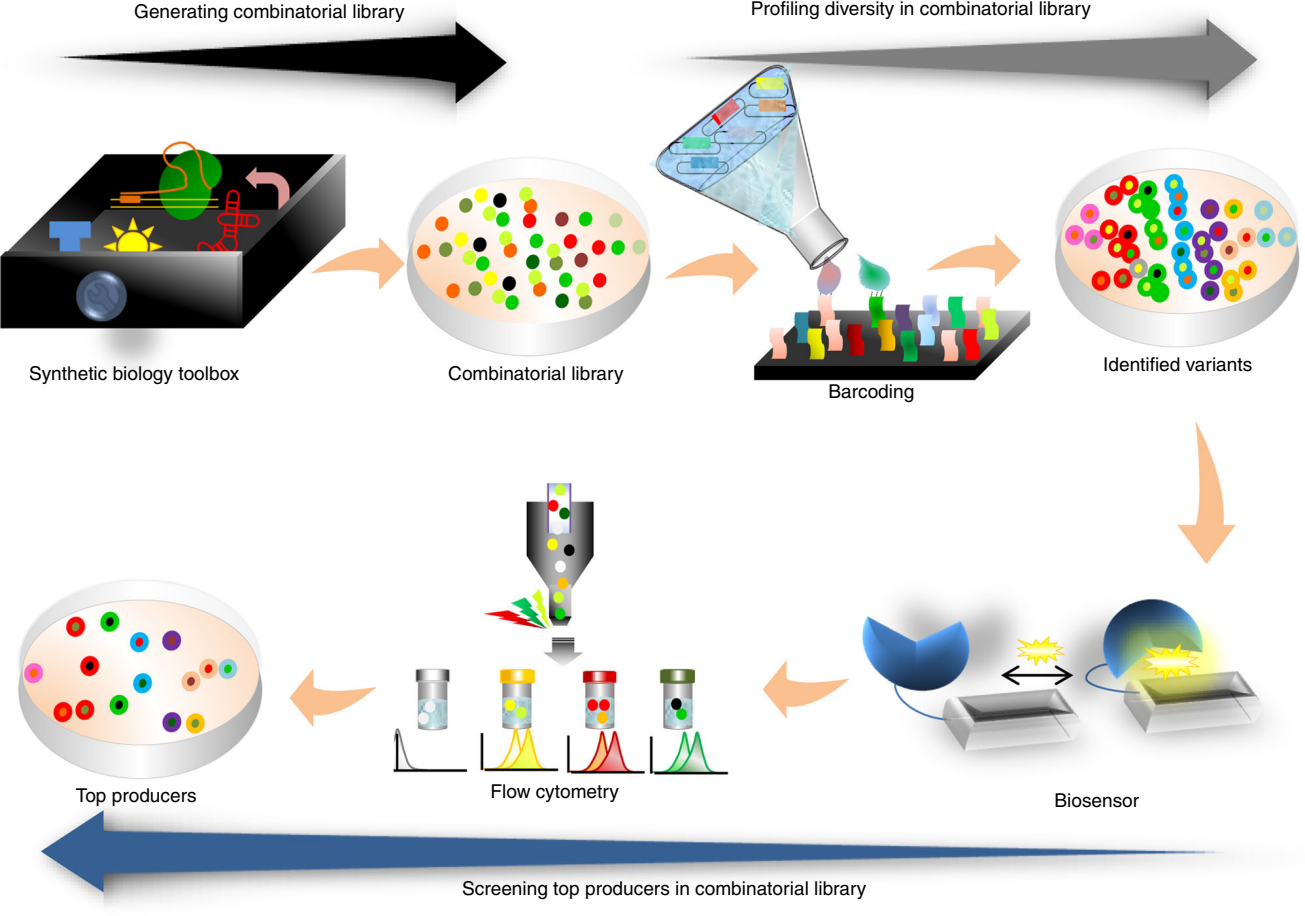
Applying synthetic biology tools toward optimized production of chemicals. Synthetic biology speeds up combinatorial optimization. DNA modification tools in the synthetic biology toolbox provide combinatorial optimization methods with various tools e.g. regulators and genome editing tools (black arrow). Barcoding allows tracking of combinatorial library members through screening steps (gray arrow). Biosensors paired with high-throughput monitoring techniques, such as flow cytometry, improve selection of library members to isolate (blue arrow).

Synthetic biology tools^41^ and design principles (Fig. 2, black arrow) are being used to accelerate development of combinatorial optimization methods. The barcoding tools^42^ can next be used to study the versatility of combinatorial optimization techniques at DNA level (Fig. 2, gray arrow). However, the identification of microbial strains in a library that produce the highest level of a metabolite of interest often remains a laborious task, mainly due to time-consuming metabolite screening techniques^39,40^. To address this issue, the genetically encoded whole cell “biosensors” and the laser-based flow cytometry technologies are combined to transduce the production of chemicals into easily detectable fluorescence signal (Fig. 2, blue arrow)^5^.

### Generating combinatorial library

**Fig. 3:**
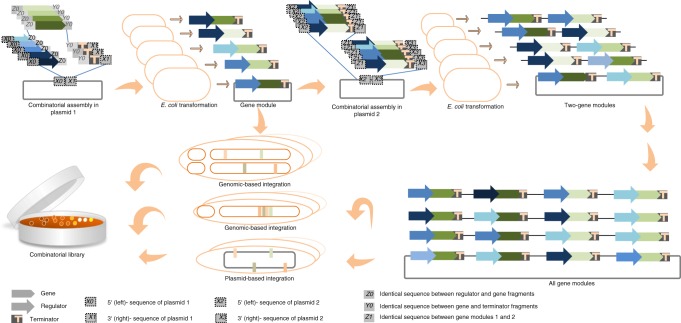
Schematic workflow to generate complex combinatorial library. Construction of a combinatorial library relies on iterative engineering cycles of one-pot assembly reactions, and amplification of assembled products in microbial cells. At the level of the assembly reaction, the reaction cocktail contains libraries of genetic elements such as promoters (blue arrow), genes (green arrow), and terminators (orange “T”). Combinatorial assembly allows assembly of all standard elements (e.g. promoters, genes, and terminators) in different combination in a single cloning step. To do this, homology sequences (for homology-based cloning method) or sequences that consist of a restriction enzyme cleavage site (for classical digestion/ligation method) at the ends of the fragments to assemble are required: *X0* and *X1* are segments upstream (left) and downstream (right) of the cloning in plasmid 1, respectively; segment *Z0* represents the 3′ region of the promoter and overlaps with the sequence upstream (left) of the gene; *s*egment *Y0* represents the 3′ region of the gene and overlaps with the sequence upstream (left) of the terminator. Thereafter, the multiple groups of gene modules of may be integrated into multi-locus of the host genome. A first combinatorial reaction cocktail is used for assembly of gene module, while a second reaction is used for generation of two-gene module from individual gene module in plasmid 2. *X2* and *X3* are segments upstream (left) and downstream (right) of the cloning in plasmid 2, respectively; and segment *Z1* represents the 3′ region of the first gene module and overlaps with the sequence upstream (left) of the second gene module. After establishing a plasmid library containing the entire pathway gene modules, the plasmid library can be directly transformed into the host or can be integrated into the genome of the host to generate stable combinatorial library variants.

Combinatorial cloning methods aim to generate multigene constructs from libraries of standardized basic genetic elements such as regulators, gene coding sequences, and terminators using a series of one-pot assembly reactions^8,39^. In Fig. 3, we illustrated a tailor-made pipeline for a complex combinatorial library generation. The workflow starts with in vitro construction and in vivo amplifying of combinatorially assembled DNA fragments to generate gene modules. Terminal homology between adjacent assembly fragments and the plasmids allows generating diverse construct in single cloning reaction. In each module, the gene expression is controlled by library of regulators^39^. To decrease the turnaround time in bioengineering projects, CRISPR/Cas-based editing strategies are implemented for multi-locus integration of multiple groups of modules into loci, whereby each group is integrated into a single locus of different microbial cells^39^. Sequential rounds of cloning enable the construction of entire pathway in a plasmid. The established plasmid can be either transformed into the host (e.g. VEGAS method^43^) or be used for single- or multi-locus integration into the microbial host genomes to generate combinatorial library (e.g. COMPASS^39^). Therefore, combinatorial optimization projects require tools and methods to assemble parts in genetic circuits, to change DNA sequences, and to integrate DNA pieces into the genome of an organism^31^. Here, we discuss two important synthetic biology tools: “advanced orthogonal regulators” and “advanced genome-editing tools”, as well as the recently established combinatorial optimization strategies.

### Advanced orthogonal regulators

Constitutive promoters are typically used to express heterologous genes in microbial cell factories, but this is often metabolically burdensome as the formation of the product competes with cell growth and proliferation^8^. Hence, auto-inducible (or self-induction) protein expression systems were established to pair growth and induction of recombinant proteins at desired time^44^. The system utilizes a cell density-based control module that allows tight regulation of the transcription of the recombinant gene; at low cell density, its expression is negligible while at high cell density expression is high. Such cell density-based control systems usually employ the quorum sensing (QS) mechanisms from *Vibrio fischeri* implemented in *E. coli*. However, these systems require the use of an extra plasmid for the production of proteins under control of the regulatory elements, which might be undesirable, particularly when multiple genes must be expressed. Another way of controlling the expression of regulators takes advantage of phage-derived anti-CRISPR proteins that allow fine-tuning the activity of dCas9-derived regulators at desired time points. The anti-CRISPR molecule inhibits the binding of Cas9 protein to DNA. This allows cells become resistant to further gene editing^45^. In another work, β-farnesene, pantothenate (a metabolic precursor of coenzyme A) was developed as a metabolic switch that effectively postpones metabolic burden until an optimal time for achieving maximal yield is reached^46^. Cultivation in media lacking pantothenate removes the growth advantage of low-producing mutants, resulting in improved production upon a scale-up to lab-scale bioreactors.

Small RNAs can also be used to control the expression of genes by RNA–DNA or RNA–RNA interactions^47^. More specifically, small RNAs can affect the chromatin structure allowing regulation of gene expression at the transcriptional level, or can affect mRNA stability post-transcriptionally or during translation. As a more sophisticated solution, orthogonal (and inducible) ATFs have been developed recently to control the timing of gene expression in various microorganisms^48^. To generate ATFs, the DNA binding domains (DBDs) of zinc finger proteins (ZFPs)^49^, transcription activator-like effectors (TALEs)^15^, and CRISPR/dCas9^9,15^ scaffolds are used. Other DBDs, including LexA^50^, SrpR^50^, PhlF^50^, TarA^50^, Bm3R1^50^, TetR^50^, auxin based degron^51^, FadR_Sa_, CarH^52^, and plant TFs^8^ may be promising alternatives. However, unlike DBDs from TALE and CRISPR/dCas9 proteins, these DBDs cannot be easily modified to target any desired genetic sequence because they need to be paired with their own specific binding site. Nevertheless, ATFs have so far rarely been used in metabolic engineering projects due to their often large molecular size or low transcriptional activity. Surprisingly, plant-derived ATFs have recently been developed as strong regulators for *S*. *cerevisiae* (10-fold stronger than the yeast constitutive and strong *TDH3* promoter)^8^. Expression of ATFs can be controlled by either exogenous chemical inducers (e.g., IPTG, arabinose)^8^ or by light of specific wave lengths^53^. However, hypersensitivity, toxic and pleiotropic effects limit the utilization of chemical inducers. An important task of future research is the identification of cost-effective inducers (chemical or others) that allow to control and modulate protein levels in response to a defined input signal in a fast-acting, tunable and robust manner. In this regard, light-based (i.e., optogenetic) systems have been developed that allow the expression of a gene of interest to an anticipated level by exposing the metabolite producing cells to short light pulses. Previous reviews have described diverse optogenetic control systems^54^. In Table 2, we list reports about different light-inducible systems, DBDs, transcription activation/repression domains (A/RDs), and the light types utilized to regulate their expression in various hosts, published since 2012. To the best of our knowledge, light-inducible systems have not yet been employed in combinatorial optimization methods. Rapamycin and its synthesized analogues are commonly used chemical inducers of dimerization (CIDs) employed by chemical biologists to place biological processes under conditional control. These CIDs bind to FK506 binding protein (FKBP) with a remarkably tight binding affinity. This FKBP-rapamycin complex then binds to the FKBP binding domain of mTOR (FRB)^55^. Such complexes have been largely employed as heterodimerization tools for small molecule switches. Unlike photoswitchable protein dimerizers (Table 1), FKBP and FRB are significantly smaller molecule photoswitches and do not need pulsed (or even constant) irradiation^55^, allowing minimum potential for phototoxicity. Considering the recent progress in the area of new light-sensing proteins^56^ and light-inducible circuit building^55,57^, light-controlled systems are more likely to be able to orthogonally regulate the expression of genes compared to chemical-dependent systems.

**Table 1:** Various light-inducible systems developed since 2012.

Light	DBD	A/RD	Photoreceptor and partner	Host	Reference
Blue	ZFP	VP16 AD	LOV and GI	Human cell	Polstein et al.^109^
Red	TALE	VP64 AD	Cry2 and CIB1	Mammalian cell	Konermann et al.^110^
Red	Gal4	VP16 AD	PhyB and PIF3	Mammalian cell	Müller et al.^111^
UV-B			UVR8 and COP1		
Blue			LOVpep and PDZ		
Red	TetR	VP16 AD	PhyB and PIF3	Plant cell	Müller et al.^112^
Blue	CRISPR/Cas9	VP64 AD	Cry2 and CIB1	Mammalian cell	Polstein et al.^113^
Blue	CRISPR/Cas9	P65 AD	Cry2 and CIB1	Mammalian cell	Nihongaki et al.^114^
Blue	LexA	VP16 AD	Cry2 and CIB1	*S. cerevisiae*	Taslimi et al.^115^
Red	TALE	VP64 AD	PhyB and PIF3	*S. cerevisiae*	Hochrein et al.^53^
Red	TALE	VP64 AD	PhyB and PIF3	*S. cerevisiae*	Hochrein et al.^57^
Blue	N terminal-T7 RNAPs	C terminal-T7 RNAPs AD	nMag and pMag	*E. coli*	Baumschlager et al.^116^
Blue	LexA	Gal4 AD	ClpX and ClpP	*S. cerevisiae*	Xu et al.^117^
Blue	Gal4	Gal4 AD	WC-1 and VVD	*S. cerevisiae*	Salinas et al.^118^
Blue	TetR	P65 AD	Cry2 and CIB1	Plant cell	Yamada et al.^119^
Green	AdoB12	VP16 AD	CarH and CarO	Plant cell Mammalian cell	Chatelle et al.^52^
Blue	ZFP	VP16 AD	CRY2 and CIB1	*S. cerevisiae*	An-adirekkun et al.^120^
Blue	NLS	dCas9 RD	LOV and α helix	*S. cerevisiae*	Geller et al.^121^

### Advanced genome-editing tools

Chromosomal pathway integration projects are classically divided into multiple steps because the rate of native double-strand breaks (DSB) followed by homology-directed repair (HDR) is not high enough to support simultaneous generation of large numbers of integrations, even in a suitable host like *S. cerevisiae*. To greatly increase the recovery of HDR-based genetic engineering events, selection markers are often included in DNA integration cassettes. Recycling the marker genes, after their initial integration into the genome, allows cells to become competent for the next round of pathway engineering, as markers can be “re-used”. The Cre-LoxP system^51,57,58^ and CRISPR/Cas9-mediated genome modification can be utilized to remove or mutate the selection marker coding sequences^59^. However, iterative rounds of engineering long pathways are time consuming.

A alternative is the CRISPR-Cas9 system (RNA-guided genome editing tool) which induce DSB and therefore allow higher efficiency in genome editing^60–63^. The combinatorial optimization method COMPASS allows the generation of a library of stable *S. cerevisiae* variants with thousands to millions of different members through only four cloning reactions followed by a one-step decoupled CRISPR-Cas9-mediated integration of the variants into the genome^39^. In a recent study, a method for the manipulation of the genome of mammalian cells was established through combining the CRISPR-Cas9 and the Cre-Lox systems^64^. In another effort, two optogenetic recombinases were developed for *E. coli*^65^. This approach uses split Cre and Flp (originally native to *S. cerevisiae*) recombinases coupled with photodimers, where blue light brings the split protein together to form a functional recombinase. However, application of DSB-mediated genome editing is limited by the cellular cytotoxicity of HDR, unwanted DNA insertions and deletions, or unwanted additional DSB. To overcome these limitations, Barbieri et al. developed eMAGE (eukaryotic Multiplex Automated Genome Engineering) to precisely modify multiple sites of a genome without an involvement of DSBs. The method utilizes synthetic single-strand DNA (ssDNA) oligonucleotides targeting the lagging strand of the desired gene(s) in the replicating chromosome. Lambda (λ) phage Red Beta ssDNA annealing protein facilitates annealing of the ssDNA oligonucleotides to the lagging strand of the targeted DNA during DNA replication at the replication fork in *S. cerevisiae*^66^. Genome editing area of research can (i) shorten the gap between genome engineering and combinatorial library construction and (ii) the generation of diverse cell variants.

### Combinatorial optimization strategies

A multi-gene pathway can be combinatorially optimized at the DNA level by altering gene copy number, at the mRNA level by controlling transcriptional output and transcript stability, at the protein lLianevel by modifying translational capacity, protein post-translational modification, protein stability, and protein co-localization, at the metabolite level by dynamic metabolic flux control and at the chassis level by engineering microbial consortia. Lian et al. developed a combinatorial strategy based on an orthogonal tri-functional CRISPR system that combines transcriptional activation, transcriptional interference, and gene deletion (CRISPR-AID) for metabolic engineering purposes in *S. cerevisiae*^67^.

The majority of the research in the combinatorial optimization area has focused on transcriptional control mechanisms as an important control point for pathway gene expression. Currently available combinatorial approaches typically employ constitutive promoters, and therefore rely on growth-coupled biosynthesis. Versatile genetic assembly system (VEGAS) exploits the native capacity of *S. cerevisiae* to perform homologous recombination and efficiently join sequences with terminal homology. Yeast Golden Gate (yGG) is used to build transcription units (constitutive promoter upstream of a pathway gene) for VEGAS assembly. In the yGG reaction, each transcription unit is assigned a pair of VEGAS adapters that assemble up- and downstream of each transcription unit; VEGAS adaptor sequences subsequently provide terminal homology for overlap-directed assembly by homologous recombination “in yeast”. In contrast, inducible regulators allow conditional control of heterologous gene expression. Only a limited number of methods employing inducible and modular transcription factor-based controllers have been established so far, including e.g. ePathOptimize^68^ and COMPASS^39^. Combinatorial approaches typically rely on plasmid-derived systems to take advantage of the simplicity of manipulation at the plasmid level. However, genomic integration of a metabolic pathway leads to higher and more stable production of a target chemical than expression of genes from plasmids^8,53^, without the need for expensive selectable markers. In general, the chosen production host “determines” the regulatory elements (promoters, terminators, RBS), codon preferences, the way enzymes (or proteins in general) are modified by posttranslational modification (e.g., phosphorylation), and the biochemical mechanisms by which enzymes or metabolites are secreted out of the cell and into the growth medium (an important aspect of synthetic biology applications). Established combinatorial pathway optimization methods for metabolic engineering are summarized in Table 2. COMPASS, the most recently established approach for combinatorial optimization in the *S. cerevisiae*, employs inducible plant-derived artificial transcription factors (ATFs) and utilizes three technical settings, (i) multi-locus integration of multiple genes into the yeast genome, when speed of strain development is prioritized, (ii) plasmid-based systems, when easy manipulation is favorable, and (iii) single genomic locus integration of multigene constructs, when predictable regulator behavior is required (as the genomic integration position affects the protein expression level)^39^.

**Table 2:** Selected recent combinatorial optimization methods.

Method	Host	Strategy	Diversification target(s)	Main features	Application
CIChE^122^	*E. coli*	Genome	Pathway copy number	RecA-mediated HR to integrate multiple copies of a pathway into the genome	Poly-3-hydroxybutyrate lycopene
Combinatorial pathway engineering using type I-E CRISPRi^123^	*E. coli*	Genome	Pro _inducible_	Based on CRISPR interference	Malonyl-CoA, 3-Hydroxypropionate
Start-Stop^124^	*E. coli*	Plasmid	Pro _constitutive,_ RBS	3-Step pathway optimization	β-Carotene
Direct combinatorial pathway optimization^125^	*E. coli*	Plasmid	Pro _constitutive_	Based on SSA and GG assembly methods	Lycopene
Combinatorial pathway engineering^126^	*E. coli*	Plasmid	Pro _constitutive_, Protein	Based on GG assembly	FR900098
EcoFlex^127^	*E. coli*	Plasmid	Pro _constitutive_, RBS, Terminator	4-Step pathway assembly	Violacein
CIDAR MoClo^128^	*E. coli*	Plasmid	Pro _constitutive_ RBS, Terminator	3-Step pathway assembly	RFP and GFP fluorescent proteins
OLMA^129^	*E. coli*	Plasmid	Pro _constitutive,_ RBS. Protein	Overhangs of short oligo-linkers bridge the fragments and the receptor vector in a single GG assembly reaction	
ePathOptimize^68^	*E. coli*	Plasmid	Pro _inducible_	3-Step pathway assembly	Violacein
One-step combinatorial optimization^130^	*E. coli*	Plasmid	Pro _constitutive,_ RBS, Protein	Based on SSA and GG assembly methods	GFP fluorescent protein
MIPE^131^	*E. coli*	Plasmid	Pro _constitutive_, RBS, Protein, Terminator	ssDNA mediated λ Red recombineering for the simultaneous introduction of mutations at several sites	Riboflavin
Combinatorial GG, MoClo^132^	*E. coli*	Plasmid	Pro _constitutive_, RBS, Protein	3-Step pathway assembly using different type IIS enzymes in each step	
TIGRs^133^	*E. coli*	Plasmid	RBS	Various intergenic regions within operons containing multiple genes	Amorpha-4,11-diene
N-terminal coding sequences–based optimization^134^	*Bacillus subtilis*	Plasmid	NCS of gene	96 rationally selected NCSs	Nutraceutical N-acetylneuraminic acids
CRISPR-assisted OAPS^135^	*B. subtilis*	Genome	Promoter, Post-transcriptional modification	CRISPRa and CRISPRi single master regulator in combination with promoter mutants	Amylase BLA
Combinatorial optimization^100^	*S. elongatus* sp. PCC 7942	Genome	5’-UTR	Integration of gene expression cassette into genomic neutral sites	2,3-Butanediol p, Acetoin
COMPASS^39^	*S. cerevisiae*	Plasmid, Genome	Artificial TF _inducible_, Copy number_,_ CDS homologues	3-Step pathway optimization	β-Carotene, β-Ionone, Naringenin
GG-based combinatorial optimization	*S. cerevisiae*	Genome	Pro _constitutive_, Terminator_,_ Linkers	Based on GG assembly and Cas9-mediated integration into ade2 locus	Linalool, Geraniol
CRISPR-AID^67^	*S. cerevisiae*	Genome	Pro _constitutive_	3-Step pathway optimization	β-Carotene
VEGAS^43^	*S. cerevisiae*	Plasmid	Pro _constitutive_	3-Step pathway assembly	β-Carotene, Violacein
COMPACTER^136^	*S. cerevisiae*	Plasmid	Pro _constitutive_	One-step assembly	Isobutanol, Mevalonate
CombiSEAL^76^	Human cells	Genome	Engineering proteins	Combinatorial assembly of barcoded protein variants	Opti-SpCas9 and OptiHF-SpCas9, with superior genome-editing efficiency

*BS* binding site, *CDS* coding DNA sequence, *GG* Golden Gate, *NCSs* N-terminal coding sequences, OAPS oligonucleotide annealing-based promoter shuffling, *Pro* promotor, *RBS* ribosome binding site, *SSA* single strand assembly, *TF* transcription factor, *UTR* untranslated region.

Up to this point, we have highlighted the power of combinatorial optimization in order to facilitate expressing heterologous pathways in an in vivo system. The in vitro system relies on designing functional biological systems from scratch. Recently a straightforward in vitro method to smartly generate DNA library construction has been developed called “in vitro SCRaMbLE system” (an abbreviation for Synthetic Chromosome Rearrangement and Modification by LoxP-mediated Evolution) enabling optimization of the biosynthetic pathway flux via rearranging relevant transcription units^69^. This system uses Cre recombinase and purified DNA encoding multiple *loxPsym* sites mixed in a tube. Wu et al.^69^ demonstrated two strategies using the “in vitro SCRaMbLE system” for pathway optimization: (i) The top-down method that consists of a purified DNA plasmid encoding multiple *loxPsym* sites. Addition of Cre recombinase to the reaction allows generating a library of SCRaMbLE DNA. (ii) The bottom-up system that employs an “acceptor vector” and a pool of “donor fragments” flanked by *loxPsym* sites. With the addition of Cre recombinase to the tube, donor fragments are randomly assembled into the acceptor vector. Therefore, a library of diverse plasmids is generated. The plasmid library can be subsequently transferred to a host strain for identification of genotype of each individual derivative. The in vitro SCRaMbLE system basically provides optimization in ways that the in vivo system cannot accomplish. However, application of combinatorial optimization strategies in in vitro production of proteins (cell-free protein synthesis (CFPS))^70^ or ex vivo (experimentation performed on tissue from an organism in an external environment)^71^ systems have not been studied yet. Such systems are typically used to produce toxic and/or large protein complexes, as there is no need to maintain cell viability. Combinatorial optimization strategies can be implemented to generate various combinations of genes and regulators in one cloning reaction cocktail. The in vivo and ex vivo systems require cellular lysate, transcription and translation factors^72^ to express different subunits of a large protein complex from the complex library of linear or plasmid DNA.

### Profiling diversity in combinatorial library

Linking the diversity within a combinatorial library at the DNA level to the production level of various members of combinatorial library can guide the debugging process to achieve the desired performance. However, the ability to capitalize on the combinatorial library diversity is limited by the number of individuals that can be tracked and assessed. To ascertain the diversity present in a combinatorial library, sequencing analysis is often used. While the identification of DNA diversity in small library can be achieved by Sanger sequencing, next-generation sequencing (NGS) has opened new ways to tackle complexity quality assessment of DNA diversity in a large library. Nevertheless, NGS is not suitable for identifying and characterizing ATFs implemented in COMPASS^39^ due to presence of repetitive sequences (e.g. ADs, minimal promoters, and terminators) in various ATFs.

Mapping the effects of protein mutation on its activity through employing high-throughput protein engineering methods or highly parallel mapping of genes to traits facilitated by multiplex DNA synthesis are now available and allow testing of a whole (or major fraction of) library population^73^. Microarray technology combined with molecular barcoding has been used to enable parallel tracking of genetically different individuals^74^. For example, trackable multiplex recombineering (TRMR) allows evaluation of thousands of definite genetic modifications in *E. coli* within a week. To do this, synthetic DNA cassettes with associated molecular barcodes were integrated into the *E. coli* genome, producing thousands of variants. Barcode sequences and microarrays were then used to compute population dynamics^42,75^. In another example, a pooled library of barcoded labelled mutants of *Streptococcus pyogenes* Cas9 (SpCas9) nuclease were easily tracked by high-throughput short-read sequencing^76^.

We can extend the design principle of these methodologies to combinatorial libraries. Each DNA cassette encoding the individual fragment (e.g. regulator, gene of interest, terminator) can conceivably be labeled with a unique molecular barcode. Thereby, thousands of fragment combinations can be tracked by counting the frequency of the molecular barcodes. Moreover, sequencing short barcode fragments can significantly reduce the time and cost for sequencing regulators and downstream genes in individual isolates. However, to track the evolving genetic heterogeneity in a population of growing production cells, sequencing individual short barcode fragments is not sufficient and more advanced sequencing techniques such as “deep DNA sequencing” are required^47^. Deep sequencing, i.e. sequencing a genomic region multiple times, is an NGS approach that can be applied to track the genetic heterogeneity in a library of cell isolates. Höllerer et al. introduced a wildly applicable DNA-based phenotypic recording approach to generate huge datasets linking regulators to quantitative functional readouts of high precision, only relying on sequencing short tag DNA elements^77^. The technique implements a site-specific recombinase, a regulator that controls recombinase expression, and a DNA substrate modifiable by the recombinase. Both regulator sequence and substrate state can be determined in a single sequencing read, and the frequency of modified substrates amongst constructs harboring the same regulator presents the quantitative effect of regulator (transcriptional output of regulator) on recombinase expression. Using next-generation sequencing, the quantitative expression effect of large library of regulators can be quantified in parallel. As a proof of principle, this approach was applied to record translation kinetics of more than 300,000 bacterial RBSs, collecting over 2.7 million sequence-function pairs in a single experiment. However, resolution of real genetic diversity in such a population can be an issue. Very recently, Askary et al. presented Zombie system for image-based readout of DNA barcodes^75^. In this system, phage RNA polymerases transcribe genomically integrated barcodes in fixed cells. The transcript RNA is then detected by fluorescent in situ hybridization. Single-nucleotide differences between barcodes are recognizable on the basis of the relative signal intensity of competing match and mismatch probes.

### Identification of top producer in combinatorial library

Selection of strains with the highest product yield from a library with huge genetic diversity is a serious bottleneck. Formation of colored products that make microbial colonies screenable is an option. However, most chemicals are not colorful and their detection requires other methods. In that case, direct quantification of product titer using low-throughput gas or liquid chromatography analysis is typically used despite being time-consuming. Methods based on spectroscopic enzymatic assay analytics are alternatives; however, they also have limited throughput. A more sophisticated solution is utilizing biosensor circuits to assay the target ligand at the single-cell level and translate their concentrations into more quantifiable signals. Biosensors are paired with high-throughput approaches, including flow cytometry and microfluidics, which allow isolation of variant cells with the phenotype of interest.

**Fig. 4:**
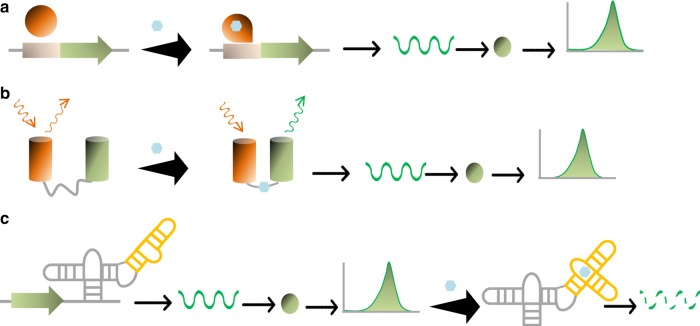
Diverse biosensors used for screening combinatorial libraries. **a** The conformation of transcription factor (TF, orange oval) changes to active form upon binding the target ligand (blue octagon). When activated, the TF binds to its binding site (light orange square), upstream of a fluorescent reporter gene, to induce production of a fluorescent reporter protein (green oval) that is detected by flow cytometry. **b** FRET sensors comprised of a donor-acceptor fluorophore pair. Ligand is sandwiched between the two donor (orange cylinder) and acceptor fluorophores (green cylinder). Therefore, a conformation of FRET is changed that allows detecting the fluorescent signal by flow cytometry. **c** Correctly folded aptamer structure of riboswitch (orange–gray structure) allows transcription of fluorescent reporter gene (green arrow). The production of fluorescent protein (green oval) is detected by flow cytometry. In presence of ligand (blue octagon), the secondary structure of riboswitch device is changed. Consequently, transcription of its fluorescent reporter gene is inhibited.

Only TF-^5,78,79^ (Fig. 4a), fluorescence resonance energy transfer (FRET)-^80^ (Fig. 4b), and RNA-based biosensors-^3,11,81–83^ (Fig. 4c) based biosensors are commonly utilized for metabolic engineering. TF-based biosensors, natural sensory proteins that regulate gene expression in response to environmental signals, employ the host’s transcription system to drive the expression of a reporter gene. Skjoedt et al.^84^ (2016) implemented systematic engineering of multiple parameters to establish a general biosensor design in the yeast *S. cerevisiae* based on metasbolite binding transcriptional activators from the superfamily of LysR-type transcriptional regulators (LTTRs) of prokaryotes. They next used the biosensors to screen cells producing different level of naringenin or *cis*, *cis*-muconic acid. The designed biosensor output correlated with the production of metabolite. LTTR-based biosensors have already shown their utility to screen top producers: in one example they were used for screening 0.0000025% of the theoretical complexity of Narion (β-ionone and naringenin co-producer) library. Rogers et al. attempted to evaluate the tuning of four genetically encoded protein sensors that respond to acrylate, glucarate, erythromycin and naringenin on either low-copy or high-copy plasmids^5^. Higher number of the intracellular gene constructs ensured higher dynamic range as well as rapid response to the target ligand^5^. However, TF-based biosensors typically have relatively high background noise, and a lot of current research focuses on addressing this shortcoming. FRET biosensors encompass a pair of donor and acceptor fluorophores. A ligand-binding peptide is sandwiched between a pair of donor and acceptor fluorophores, and ligand binding is observed via the FRET change. A set of FRET biosensors, based on three pairs of donor/acceptor, carboxyfluorescein (FAM)/Boron dipyrromethene (BODIPY)^85^, Nitrobenzoxadiazole (NBD)/nonsteroidal dye (Nile red)^86^, and coumarine/NBD^86^, was developed for real time monitoring of acid sphingomyelinase at high sensitivity and with high spatial resolution. The FRET biosensor is selectively cleaved by sphingomyelinase that leads to significant increase in fluorescence of the fluorescein FRET donor^85^. FRET biosensors have high orthogonality, high temporal resolution, and relatively easy construction^80^. The RNA-based biosensors include RNA riboswitches and RNA Spinaches. In the case of RNA riboswitch-based biosensors, the regulatory domain of an mRNA selectively binds to a ligand resulting in a structural change to the response domain that regulates translation of its encoded protein. Abatemarco et al. developed RNA riboswitch-based biosensor to detect the production of tyrosine and streptavidin concentrations in *S. cerevisiae* library. The aptamer is co-encapsulated with a member of a yeast library followed by incubation to produce the molecule of interest and development of a fluorescence signal. The picoliter droplets flow through a microfluidic device allowing sorting based on fluorescence^87^. In comparison to TF based biosensors, their kinetics are faster because the RNA has already been transcribed and so offer faster responses to target metabolite. They also do not rely on protein-protein or protein-metabolite interactions. This allows for more targeted engineering of the aptamers (the ligand binding domain) and the expression platforms. The development of biosensors that allow in vivo evaluation of any desired product of metabolic engineering would be an absolute boon for the field. A step in this direction is the systematic evolution of ligands by exponential enrichment (SELEX) approach, which can be, at least in principle, used to generate artificial riboswitch-based biosensors (single-stranded RNA aptamer) for any target metabolites. Using SELEX, metabolite-responsive riboswitch-based biosensors are developed from a library of nucleic acids. The selected riboswitch-based biosensors are, subsequently, employed to detect and report the metabolite signal^88^.

Very recently, a high-resolution methodology for the cell-specific RNA labeling was established based on nucleoside–enzyme pair^89^. The small molecule–enzyme pair consists of uridine/cytidine kinase 2 and 2′-azidouridine, where 2′-azidouridine is only incorporated in cells expressing uridine/cytidine kinase 2. This pair can be used to purify and track RNA from specific cellular populations.

## Computational modeling to advance combinatorial optimization

**Fig. 5:**
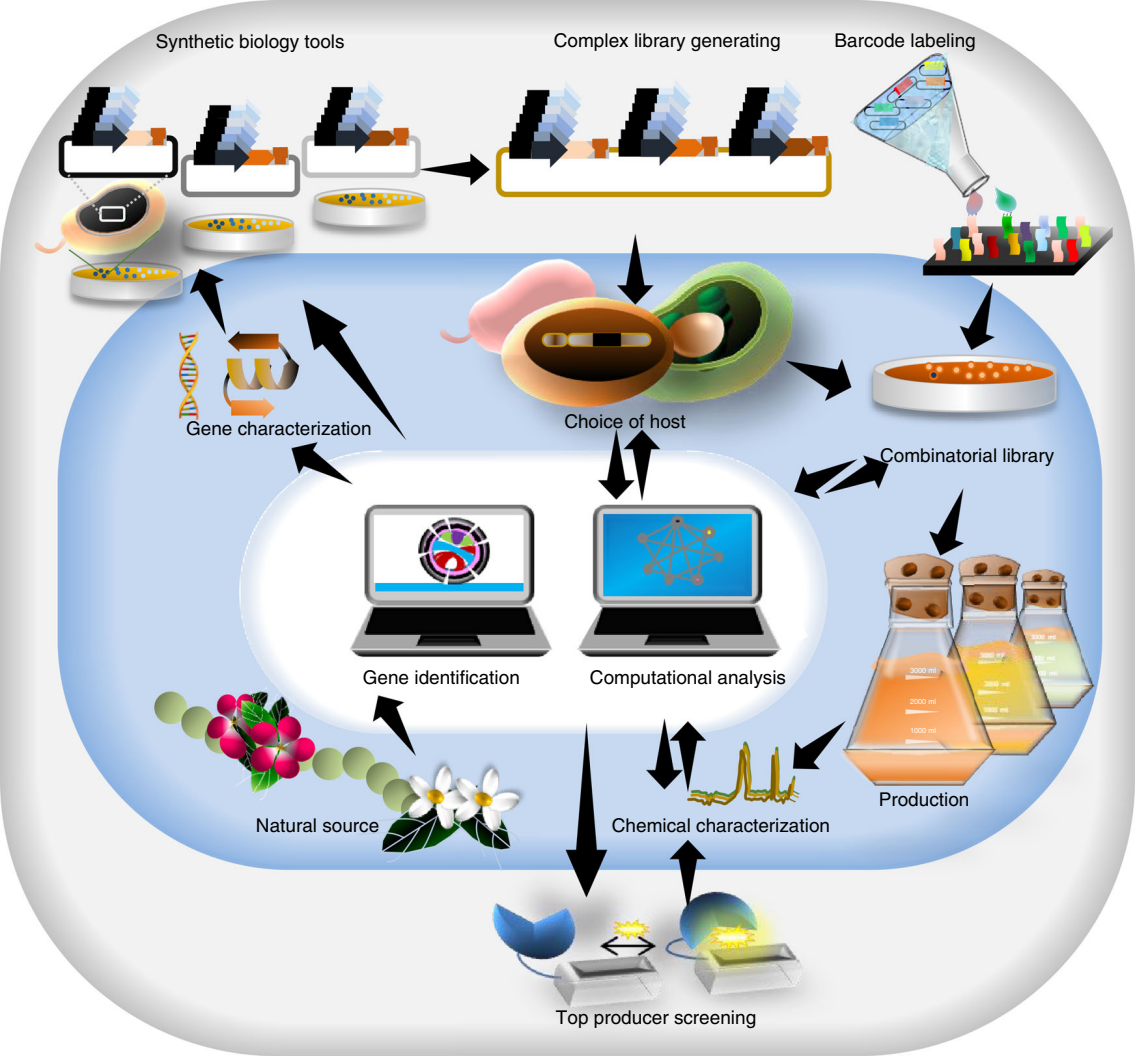
Schematic overview of computational design and evaluation to achieve optimal performance. Synthetic biology tools are used to establish combinatorial optimization methods. The generated library is profiled using barcoding tools and biosensors allow to screen top producers within the library (gray area). Nature is a vital source of identified nutrients and pharmaceuticals. Metabolic engineering applies synthetic biology tools to produce compounds of the characterized biosynthetic pathway in a desired host (blue area). The production of certain compounds can be optimized using combinatorial optimization strategies. The data obtained from combinatorial library and its pre-characterized modules are computationally integrated to establish mathematical models to support the early design steps for chosen host on the basis of genome-scale metabolic modelling. The computational data suggest which synthetic pathways are the most promising in a given target organism and which host pathway genes need to be upregulated or be silenced based on knowledge of how different cellular subsystems work together. The best producers in combinatorial libraries can provide detailed information to feed into models that aim to uncover principles of how synthetic circuits behave in host systems. Blue arrows, regulators. Orange squares, CDSs. Brown “T”, terminators.

Metabolic engineering efforts aim to optimize the cellular processes for production of a compound of interest in host of choice (Fig. 5, blue area). To bridge actual and desired function of enzymes and pathways, the complexity of genome-wide metabolism necessitates directed evolution approaches^90^. The directed (or adaptive laboratory) evolution improves the natural evolution process by (i) randomization of mutation or recombination and (ii) accelerated screening rate of positive strains^90^. It uses different databases, libraries of tools and conditions to generate the optimal production rate of a desired compound. Mathematical models and computational simulation could be applied as powerful tools to understand and predict the behavior of the biological systems.

The multi-level omics data obtained from different databases for pre-characterized synthetic biology parts and modules are computationally integrated to establish mathematical models to support the early design steps of a system. The computational design (Fig. 5, white area) approaches allow connecting reactions obtained from various databases; for example, enzymes can often recognize molecules that are similar to their natural precursor. Next, computational design approaches calculate all possible metabolic pathways to achieve wanted product. Although the computational modeling of synthetic metabolic networks has improved tremendously, the experimental data obtained from modeled synthetic pathways is not always in agreement with the best modeled pathways suggested by computational modelling (in even very well characterized organisms). In fact, to generate data for computational model building, better high-throughput techniques for analysis of the cellular transcriptome, proteome, and metabolome are still necessary. Some combinations of pathways may include unrealistic reactions or too long pathways. Moreover, dynamic models usually do not cover a large number of reactions commonly involved in a pathway. In such a case, the counted pathways required to be evaluated. Recently, Carbonell et al. built and demonstrated an automated Design–Build-Test–Learn (DBTL) pipeline to computationally select the most promising parts, e.g. enzyme coding regions and RBSs, for assembly, allowing significantly more manageable experimental screening effort^91^. The DBTL pipeline led to a 500-fold increase in titer of the flavonoid (2*S*)-pinocembrin from screening of only 65 variants out of more than 23,000 possible combinations. Although this system was developed for *E. coli*, in principle it should be applicable for synthesis in other chassis. RBS Calculator allows the prediction of the translation initiation rate of internal start codons, particularly to minimize undesired expression in *E. coli*^19,92^.

Several toolboxes are now available to integrate transcriptomics, proteomics, fluxomics and metabolomics data. Tn-Core allows automatic integration of Tn-Seq data in addition to RNA seq data to generate context specific models. This provides a “systems-level” view for metabolic engineering aims^93^. Principal component analysis (PCA) was recently implemented to evaluate various engineered strains for metabolites that contribute to acetol formation using metabolomics datasets. This data helped identify NADPH regeneration as the bottleneck for efficient acetol biosynthesis^94^. However, few laboratories work concurrently on both experimental and computational aspects of metabolic engineering, a fact that impedes the robust information exchange between these domains that is necessary for the highest productivity. RiboLogic tool was developed for designing riboswitches that are responsive to RNA inputs, as well as small molecule ligands^3^. RiboLogic algorithm was applied to design 286 switches that modulate MS2 (bacteriophage coat protein) binding of flavin mononucleotide, tryptophan, theophylline, and miR-208a, a 22-nt miRNA implicated in cardiac hypertrophythe.

The use of selective marker genes as biosensors, links the host production performance with growth rate. Therefore, biosensors make possible to study directed evolution of the microorganism or the entire biological network (Fig. 5, grey area). Additionally, combinatorial optimization, by rapid generating thousands to millions constructs, makes it possible to circumvent insufficient knowledge of in vivo reactions. This allows achieving the best compromise (solution) among conflicting pre-defined subjects. In other words, the best performer achieved from a combinatorial library can guide the debugging process to achieve the wanted performance. This data combined can be used to design a computational model for generation of optimal constructs through mathematical data to minimize or maximize wanted functions out of required subjects. Finally, computational methods can suggest possible synthetic pathways that are predicted to perform the best in a target organism and the performance of the chosen synthetic pathways is evaluated in the laboratory. Overall, results achieved from combinatorial optimization and integration of these data into available computational predictive models, can provide better understanding of the whole cellular system^95^. Höllerer et al., implemented next-generation sequencing to assess the quantitative expression effect of extremely large sets of RBSs^77^ . They expanded from these large-scale datasets using a novel deep learning approach that combines ensembling and uncertainty modelling to predict the function of untested RBSs with high accuracy. The data achieved from DNA-based phenotypic recording supports deep learning databases and, therefore, provides a major advance in our ability to predict quantitative function from genetic sequence.

## Future perspective of combinatorial optimization

**Fig. 6:**
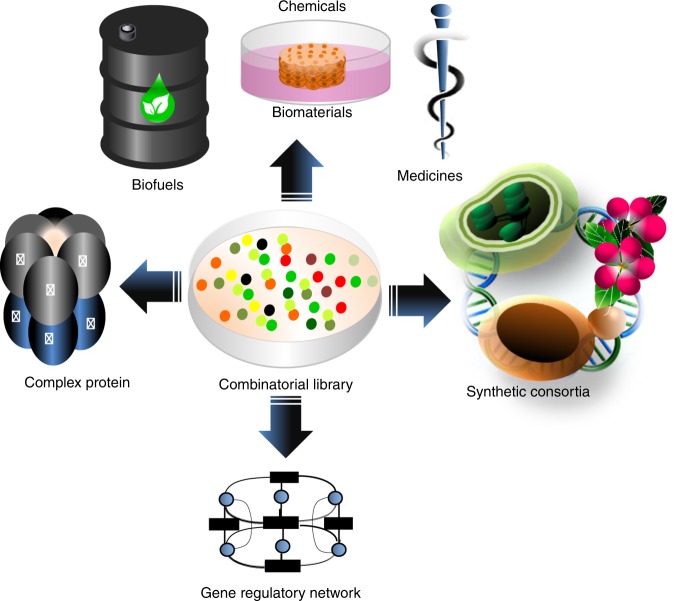
Application of combinatorial optimization strategies. One main aim of synthetic biology is development of microbial strains able to optimize and maximize yield and productivity of target chemicals, e.g. biofuels, biomaterials and, medicines, or multi-subunit cellular complexes that can be facilitated by applying combinatorial optimization approaches. Another interesting goal of synthetic biology is engineering sophisticated GRNs to expose the genetic architecture of complex traits and diseases. Smartly designed combinatorial libraries can generate huge number of GRN variants, where the optimal expression level of regulators of networks can be monitored. To overcome limitations regarding the transferability and expression of all involved systems in one chassis, a promising alternative solution is to focus on parallel optimization of metabolic pathways divided among different cells in synthetic microbial consortia^107,108^.

Combinatorial optimization techniques can be implemented to construct gene circuits that satisfy quantitative performance, something that has been a long‐standing challenge in synthetic biology (Fig. 6).

In the past 30 years, a number of microbial processes have been developed for the production of such high-value chemicals including taxol^33^, strictosidine^96^, opioids^97^, and cocoa butter-like lipids^98^. Until now, production of only a few compounds, such as 1,3-propanediol^23,99^, 1-4-butanediol^26,100^, β-farnesene^45,101^, and amorphadiene^102^ has reached commercial scale. In order to achieve fermentation titres of 25 grams per litre of artemisinic acid, plant-derived heterologous genes were engineered into *S. cerevisiae*. Furthermore, an efficient and scalable chemical process for the conversion of artemisinic acid to artemisinin using a chemical source of singlet oxygen was developed^102^. In another study, the heterologous genes encoding biosynthetic enzymes of the taxol biosynthetic pathway, isoprenoid pathway, and the regulatory factors to inhibit competitive pathways were introduced into the yeast *S. cerevisiae* to produce 33 milligram per liter of taxadiene^33^. In both projects, the yeast promoters were used to express the engineered enzymes on a high-copy plasmid. Inducible orthogonal regulators^8^, and combinatorial optimization approaches allowing to genomically integration of pathways genes^67^ can pave the way to producing natural and novel chemicals for new applications such as “non-natural” variants of thaxtomin phytotoxins^73^ and new peptides with “designer” functional properties. Protein production for biotechnological and medicinal applications is a multibillion-dollar market. Chemical synthesis of protein molecules is prohibitively expensive. Several expression systems ranging from bacterial hosts to mammalian cells have been established^24^. However, only 25 of them have been produced at a bioreactor scale^24^. In fact, biological production of complex proteins is facing a series of challenges, such as the need for balanced expression of multiple subunits of large proteins. Combinatorial optimization strategies allow expression of various subunits of protein complex in different ratios and, by doing so, facilitate the correct assembly of multi-subunit complexes (i.g. cytochromes, catalases, P450 enzymes, and carboxysomes).In particular, improvements in synthetic biology have made possible to study gene regulatory networks (GRNs) in a simplified setting amenable for exact experimental controls.

For example, researchers constructed mutual inhibition toggle between TetR and LacI inhibitors^103,64^, bistable circuit of autoactivation^104^, or logic XOR and AND gates^105^. Combinatorial optimization coupled with biosensor techniques can facilitate generation of different possible GRNs, whose output can be measured and compared by placing a reporter gene downstream of the last regulator of the network and the output quantified by flow cytometry. This paves the way to understand regulatory relationships among genes and identifying key regulators and bottlenecks in GRNs.

There is no super host that is best for the production of all target molecules^106^. *S. cerevisiae* is a better production host than *E. coli* for production of amorphadiene, and β-farnesene due to less toxicity of pathway intermediates^101,102^, but producer populations were compromised by a high percentage of nonproducer mutants^47^. Hypothetical scenario to handle especially complex synthesis pathways is to divide them into pieces and design a different synthetic microbial host for each piece. The engineered microbes can then be grown together in consortia^107,108^. This can be substantially mitigated by utilizing combinatorial design principles to optimization multiple portions of the complex pathway (across multiple hosts) in parallel.

## Future directions

Synthetic biology projects often need tunable expression of various combinations of genes to achieve an optimal output. Ideally, computational models will ultimately facilitate the forward-engineering of many parts to achieve desired function. However, until then the best alternative is to rapidly generate and accurately characterize as many new variants as possible. Therefore, smartly designed combinatorial optimization methods for the empirical balancing of metabolic pathway gene expression is of interest to a broad range of synthetic biologists. The workflow described in this review has the potential to become an enabling standard for researchers seeking to achieve optimal output while minimizing experimental effort in bioengineering projects. We expect that future developments in combinatorial optimization will still be limited by a lack of tools to screen the best producers from such a library. To continue advancing in our ability to engineer living organisms for high-level production of desired compounds using combinatorial optimization methods, we will require high-throughput screening techniques. Therefore, an interesting research direction would be a generalized biosensor construction framework for targeting diverse molecules. Ongoing advancements in developing riboswitch-based biosensors may become a larger part of the solution.

In this review, we discussed how DNA barcoding methodologies have significantly improved in recent years, enabling the computation of population dynamics^42,75^ or tracking of mutants of Cas9 nuclease library^76^. Such techniques however have yet to be applied for tracking of combinatorial library members. In fact, we can envision the use of the design principle of these methodologies to combinatorial libraries to identify the DNA elements combined in libraries of million circuits.

Combinatorial optimization in synthetic biology encompasses a number of different techniques and disciplines that include molecular biology, analytical and protein chemistry and computational modelling. Progress in this field is emerging from a close interplay between bioinformaticians, chemists, and synthetic biologists.
